# *Moringa oleifera*: An Unknown Crop in Developed Countries with Great Potential for Industry and Adapted to Climate Change

**DOI:** 10.3390/foods10010031

**Published:** 2020-12-24

**Authors:** Carla Trigo, María Luisa Castelló, María Dolores Ortolá, Francisco José García-Mares, María Desamparados Soriano

**Affiliations:** 1 Institute of Food Process Engineering for Development, Universitat Politècnica de València, Camino de Vera s/n, 46022 Valencia, Spain; cartrigu@etsiamn.upv.es (C.T.); mdortola@tal.upv.es (M.D.O.); 2 Department of Hydraulic Engineering and Environment, Universitat Politècnica de València, Camino de Vera s/n, 46022 Valencia, Spain; fjgarcia@gmf.upv.es; 3 Department of Plant Production, Universitat Politècnica de València. Camino de Vera s/n, 46022 Valencia, Spain; asoriano@prv.upv.es

**Keywords:** *Moringa oleifera*, agronomic requirements, nutritional profile, climate change

## Abstract

*Moringa oleifera* is originally a tropical crop with a fast development, little known in developed countries but cultivated since ancient times. It can adapt to regions affected by climate change, such as the Mediterranean basin, since it is a crop with a great resistance to high temperatures. In this study an in-depth bibliographical review was carried out by consulting different databases (Science Direct, FSTA, Scielo, Riunet, and Google Scholar) in order to find published scientific studies on the characteristics of this crop and its agronomic requirements. According to the information found, all parts of the *Moringa oleifera*, namely the leaves, pods, seeds, roots and flowers, can be used in different industrial sectors such as pharmaceutical, cosmetic, human food, animal feed, and water treatment since they have a nutritional profile rich in high biological value proteins, vitamins A and C, antioxidants, omega-3 fatty acids and minerals: calcium, iron, potassium, and phosphorous.

## 1. Introduction

The *Moringa oleifera* Lam. tree is a tropical tree that belongs to the *Moringaceae* family, which includes about 13 different species of trees [1]. However, the best known is *M. oleifera*. This crop comes from northern India and some areas of northern Europe although it is also grown in the Red Sea area and/or other parts of Asia and Africa, including Madagascar. However, this crop has been spread worldwide [2] (Asia, Africa, Central, and South America) and this fact has led to it being given different names, i.e., “benzolive tree, drumstick tree, horseradish tree, mulangay, moonga, saijhan, marango, sajna, mlonge, or Ben oil tree” [3].

The history of the *M. oleifera* tree dates back to 150 B.C. Historical evidence reveals that ancient kings and queens used *M. oleifera* leaves and fruits in their diet to maintain a state of mental alertness and healthy skin. Ancient Mauritanian warriors in India drank *M. oleifera* leaf extract on the war front and this drink was believed to be a kind of elixir that gave them extra energy and relieved them of the stress and pain suffered during the war. Eventually, these brave soldiers were the ones who defeated Alexander the Great [4].

The geographical areas where this plant was originally developed, such as the southern hemisphere, India, China and Brazil, among others, are the regions where there are currently problems of malnutrition. It is widely known that agricultural growth is particularly effective in reducing hunger and malnutrition. Thus, one harvest of a *M. oleifera* plantation’s (705 trees) total estimated leaf biomass would yield a one-day proper calorie intake for 340 adult humans (irrespective of gender) [5]. In this regard, the rich nutritional profile of *M. oleifera* can contribute to reduce poverty and hunger since its cultivation will increase workers’ incomes and generate employment for people with limited resources [6].

Moreover, the good adaptability of *M. oleifera* to different soils and climate is remarkable, along with its easy propagation [7], which is directly related to its suitable adaptation to climate change in regions where mild conditions have changed to arid ones, such as in the Mediterranean area. Hence, *M. oleifera* trees are being promoted as a dual solution to mitigate the impacts of climate change, while also providing an alternative source of income for families. The trees are easy to plant and do not require much maintenance. The planting process includes a water conservation approach that uses only 5 L of water during planting and 250–100 mL per day afterwards, decreasing as the tree grows and its roots establish [8]. In addition, the rate of absorption of carbon dioxide by the *M. oleifera* tree is twenty times higher than that of the general vegetation [9,10,11]. Therefore, the *M. oleifera* tree is useful tool in the prevention of global warming.

From an industrial point of view, the most commercialized product is the *M. oleifera* leaf powder, since it is one of the richest sources of natural iron and calcium and it is considered a multivitamin supplement with also high amounts of amino acids, among other nutrients [12]. For that, different drying methods have been studied such as microwave combined with hot air method, oven or convection drying, solar drying, and shadow drying [12,13]. Besides, *M. oleifera* is one of the most important plant genera with several economic values. The genus is well known for its multiplicity of uses. The leaves are used as nutrition supplements, seeds used for water purification, the oil as a biofuel, the trunk as gum producer, the flowers as source of honey, and every part of the plant can also be used for therapeutic purposes [2].

This review aims to detail the origin of the *M. oleifera* crop and its expansion, the botanical description of this plant, the growing conditions of the crop, the uses and benefits of its different parts, nutritional aspects, and medicinal properties. More than 80 found articles published mostly in the last 20 years have been consulted to carry out this review.

## 2. Botanical Description of the Crop and Its Growing Conditions

*M. oleifera* can be taxonomically identified according to the most up-to-date classification [6] of APG IV (Angiosperm Phylogeny Group), which is based on phylogenetic criteria. The taxonomic classification would be as follows:

Class *Eudicotyledoneae*
Subclass *Magnoliidae*
Clado *Malvidae*
Order *Brassicales*
Family *Moringaceae*
Genus *Moringa*
Species *Moringa oleifera* Lam

According to the APG IV criteria [14], the *Moringaceae* family is now part of the order of the *Brassicales*, where species such as radish and cabbage are found. The papaya family (*Caricaceae*) is the closest to the *Moringaceae*, sharing the characteristic of having glands at the apex of the petiole [15].

*M. oleifera* can be grown by direct seeding, transplanting, or using stem cuttings [16]. It should be noted that this crop is relatively easy to grow since it is spread by sexual and asexual means and it has a low soil nutrient and water demand [17]. Optimal conditions for *M. oleifera* growth are in the warm and semi-arid tropics as it is a very drought tolerant crop growing with rainfall of 250–3000 mm per year and at altitudes below 600 m [18]. However, it should be noted that its growth has been recorded at 2000 m altitude. It tolerates a wide range of environmental conditions and poorly fertile soils, even withstanding high temperatures, draughts, and light frosts [19]. The optimum temperature range is 25–35 °C and it can even withstand 48 °C for a limited period of time [20]. The *M. oleifera* tree develops its maximum productive potential in well-drained sandy or sandy loam soils. It is also able to tolerate clayey soils but not the accumulation of water for prolonged periods of time because it would cause a decrease in growth [21]. It is an extremely fast growing tree; thanks to the high yield of the crop in just three months there is a substantial growth. Normally its growth varies from 5 to 10 m high [22].

In tropical areas with rainfall spread throughout the year, this plant has constant flowering, whereas in dry tropical areas, there are two pod harvests per year. Thus, in Spain, there is only one harvest of pods between August and September [23]. As for the harvesting of the leaves, due to the high production yield, 3 to 5 cuts may be made per season.

In areas where *M. oleifera* is not originally grown, this crop is beginning to be introduced since there is a growing interest in expanding the consumption of this vegetable due to its many possibilities of being included in a healthy diet and also because it could be considered a potential crop for combating the global climate change. In the particular case of Spain, these are the limiting factors for this crop according to some studies carried out by different researchers:

- *M. oleifera* is especially sensitive to low temperatures. During the coldest months, it can withstand between −1 °C and 3 °C, tolerating short and low-intensity frosts. If the frost persists, the plant dies immediately. Consequently, low temperatures are considered to be the “exclusive” factor for the development of this plant. In the Iberian Peninsula and the Balearic Islands, December, January, and February are the months when the lowest temperatures are reached. The main mountain systems and most of the Douro Valley are unsuitable areas for the cultivation of the *M. oleifera* due to the low winter temperatures [24].
- It does not survive temperatures above 48 °C [20]. No area in Spain reaches this average temperature during the summer months (June, July, and August).
- If the average temperature exceeds 8 °C, the risk of light frost is low, so the plant could survive, although it would not begin growing [25].
- The plant needs high daily average temperatures between 25–35 °C to have an optimal growth and a high production of pods and leaves, resulting in its most cost-effective cultivation [24].
- For isohyets, the limit values are 300 mm and 500 mm [24].

Furthermore, a Japanese research report [11] has shown that the carbon dioxide absorption rate (CO_2_) of the *M. oleifera* tree is twenty times higher than that of vegetation in general. The *M. oleifera* tree has great potential not only to store carbon but also to improve the livelihoods of many smallholders. Therefore, as discussed above, planting this tree in different parts of the country could mitigate the effects of climate change [10].

To sum up, numerous researchers have concluded that the *M. oleifera* tree is a very versatile tree with rapid growth and good adaptation to adverse weather conditions. Therefore, this crop can be a good alternative to intensive cultivation to face the current battle against climate change.

## 3. Parts of the *M. oleifera* and Their Composition

**Figure 1:**
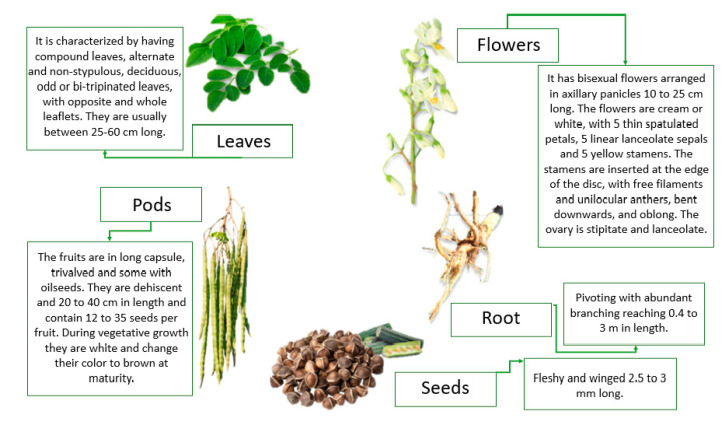
Parts of the plant and its main characteristics. (Sources: [15,27,28,29]).

*M. oleifera* has multiple uses because all parts of the tree are edible. In addition, the most surprising aspect is its exceptionally high nutritional value [26]. In Figure 1, their morphology and main characteristics are shown.

In the case of leaves, which have a high morphological similarity to ferns, according to Oyeyinka et al. [30], they contain the greatest amount of nutrients compared to other parts of *M. oleifera*, especially in terms of protein content (19–29%). In addition, they are excellent as a source of vitamin E, vitamin A (four times more than the content of a carrot), vitamin C (in fresh leaves, the amount is seven times higher than in an orange), and vitamin B. They are also one of the best vegetable sources of minerals since their calcium content is very high for a plant (more than four times the amount of milk) and the iron content is very interesting; it becomes very useful against anemia. It also has high amounts of potassium—three times the amount of a banana—as shown in Table 1. Except for vitamin C, the nutritional value of *M. oleifera* leaf powder is higher than that of fresh leaves. This can be interesting, as dried leaves can be stored so their use is guaranteed throughout the year [31]. In many cultures of poor countries, they are often the only source of additional proteins, minerals, and vitamins. In addition, its content of fats, carbohydrates, and phosphorus is very low, which makes it one of the best plant foods [32].

*M. oleifera* flowers also serve as a good source of a wide variety of nutrients, including proteins, potassium, calcium antioxidants (α and γ tocopherol), and polyunsaturated fatty acids, leading them to be ready food or tea and dietary supplement after processed [33]. Fried *M. oleifera* flowers taste like mushroom [22].

High content of nutrition in pods and seeds of *M. oleifera* have been reported in many studies. There is about 9.98–51.80 g crude protein, 17.26–20.00 g crude fiber, 3.36–18.00 g carbohydrate, 38.67–43.60 g fat, and 3.60–5.00 g ash per 100 g *M. oleifera* seeds [34]. Pods contain abundance of dietary fiber, low content of lipid, and a reasonable amount of unsaturated and essential fatty acids, especially oleic acid [35].

## 4. Uses of the Parts of the *M. oleifera*

**Figure 2:**
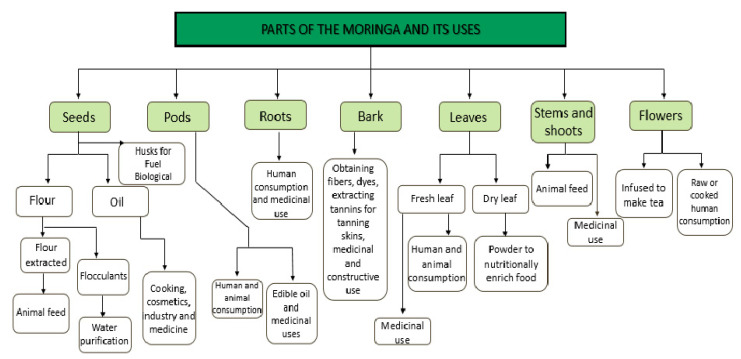
Diagram of the parts that mainly form the plant and its uses. (Adapted from [36].).

As described above, one of the uses of this plant is human consumption, mainly because of its appreciated nutritional components. Its food uses range from the formation of a main course to its use in salads or sauces. There are many plants in nature that help to restore the body balance and maintain a good health. *M. oleifera* is among the best tropical perennial vegetables from a nutritional point of view [35], as shown in Figure 2. The uses of each plant part will be explained in more detail below.

### 4.1. Seeds

The seed of this plant contains oil that can be used for cooking, in the cosmetics industry, or for medicinal purposes. The perfume extracted from the seed oil is highly appreciated by perfumers for its power to absorb and retain odors, mainly for the manufacture of deodorants [37]. The seeds also contain specific protein fractions for skin and hair care. Peptides from the *M. oleifera* seed protect the skin from environmental influences (anti-pollution) and combat premature skin aging. The seed extract is a globally accepted innovation and an active solution for hair [38]. Besides, seed flour cake is used to purify water, reducing the occurrence of waterborne diseases that cause numerous deaths in developing countries [39]. Some technological applications have been made in cookies and flour formulations for children, but the bitterness and toxicity caused by lectin (hemagglutinin) remain a limit for human consumption [40].

Furthermore, according to the literature, the oil from *M. oleifera* seeds (about 40%) has an excellent quality [41] and can be used as a raw material for the production of biodiesel [42].

### 4.2. Pods

Lobed flowers and pods are produced during the second year of growth. The pods are harvested when they are young, tender, and green, that is, as immature pods being the most valued and nutritious because they contain all the essential amino acids along with many vitamins and other nutrients [43]. The consumption of pods can be both human and animal. Immature pods can be eaten raw or prepared as peas or green beans and reported to taste like asparagus; while ripe pods are usually fried and taste like peanut. They also produce 38 to 40% of edible oil known as Ben Oil [44]. This oil is clear, sweet, and odorless—unlikely to alter the taste [22]. In general, its nutritional value resembles that of olive oil and it is worth noting that it has anti-inflammatory compounds that help relieve pain and swelling caused by arthritis, rheumatism, and gout [45].

### 4.3. Root and Bark

As for the roots, some studies [46] have shown that certain root extracts contain analgesics called *Moringin* and *Moringinine* that may play a role in their effectiveness against lumbago at appropriate doses. The roots also have a food use; they can be used as a condiment or in sauces as they have a flavor very similar to horseradish [43]. However, high consumption of roots, seeds, bark, and even leaves can be a health problem [36]. Depending on the dose and timing of consumption, alkaloids, especially those in the root and bark, can become toxic like spirochin and phytochemical bencil isothiocyanate [47]. The spirochin alkaloid can cause tachycardia at a dose of 35 mg/kg body weight and cause kidney damage at periodic doses above 46 mg/kg body weight. On the other hand, it is an effective prophylactic and antiseptic against wound infections by gram-positive bacteria, especially *Staphylococcus* and *Streptococcus*. Fibers, dyes, and tannins can be obtained from the bark for skin tanning [43].

In addition to the above, the *M. oleifera* tree can be used as windbreak to prevent soil erosion and its wood is useful as a building element for its ability to maintain heat [48].

### 4.4. Leaves

The leaves can be eaten fresh in salads, in vegetable curry, or as a seasoning (contributing to nutritional improvement especially in areas with malnutrition). They can also be cooked in soups and stews [49] or have medicinal properties. For fresh consumption, they should be harvested early in the morning and sold on the same day.

Dried leaves are interesting for preparing nutritionally enhanced foods, mixing them with legumes and cereals to try to obtain a complete protein. Older leaves should be stripped of their hard, fibrous stems as they are more suitable for dry leaf powder [50]. This powder is used to enrich food and can be stored for many months at room temperature without losing its nutritional value [51]. Its protein content (22–24%) is similar to that of soybean. Other uses would be the use of leaves as seasoning, spices, flavorings, in infusions or with medicinal functions [7].

In animal feed, the fresh leaves of *M. oleifera* have a positive effect, being as they stimulate the increase in the level of efficiency in the use of metabolizable energy by increasing microbial activity as well as a greater efficiency in the use of energy from pastures [52]. There are several experiences of including fresh *M. oleifera* fodder in the feed of different animal species such as goats, sheep, cows, birds, and pigs [34,52,53,54]. Positive effects are reported on the productive behavior of goats and sheep, increases in the quality of cow’s milk, a greater contribution of protein in pigs, and an improvement in the weight gain of sheep.

The leaves are the anatomical part of the plant, whose consumption of large doses over a long period of time means less risk to health. Specifically, it presents a lethal dose for 50% of the population (DL50) at 17.8 g/kg body weight and 15.9 g/kg body weight for the aqueous extract, which can lead to an alteration of hematological parameters and spleen hypertrophy and thymus [36]. However, no adverse effects were reported in a human study conducted with whole leaf powder at up to a single dose of 50 g or in a study using 8 g per day dose for 40 days [55].

### 4.5. Stems, Shoots, and Flowers

The dried flowers are infused for tea and have been proved to be rich in potassium and calcium [32] although they can also be consumed raw or cooked. The seeds, leaves, and especially flowers have insecticidal, larvicidal, and ovicidal activity against the vectors of the *Anopheles stephensi* and *Aedes aegypti* species [49].

Stem pulp is used for newspaper manufacturing and in textile industries [56]. The stems and shoots are used for animal feed and have medicinal effects.

## 5. Nutritional Aspects of Fresh and Dry Leaves of *M. oleifera*

In this section, a deep approach has been done by comparing the nutritional aspects of *M. oleifera* fresh and dry leaves with other foods (Table 1).

It should be noted that *M. oleifera* leaves are a very rich source of essential amino acids, which are often lacking in many vegetables [46]. Table 2 shows the main amino acids found in the dried leaves of *M. oleifera* compared to the amino acids contained in soybeans and beef. It is not usual for a vegetable to contain all these amino acids, and *M. oleifera* contains them in a good proportion, these being very useful for our body. Therefore, *M. oleifera* leaves can be of great help to people who do not get protein from milk such as vegans or vegetarians. It even contains arginine and histidine, two amino acids especially important for newborns. Most children in sub-Saharan Africa are protein-deficient and *M. oleifera* could be an extremely valuable source of food for treating malnutrition in poor countries. *M. oleifera* has been found to have a higher content of amino acids than soybeans and beef in almost all the amino acids analyzed, except for lysine, methionine, and isoleucine, where beef has a higher content.

*M. oleifera* is also a mineral reserve. Compared to mineral-rich vegetables that are usually consumed fresh such as spinach, or dried such as chia seeds, the differences in the content of these minerals can be seen in Table 3. As can be seen, spinach only exceeds the fresh *M. oleifera* leaf in terms of iron and potassium. As for the dried *M. oleifera* leaf, it far exceeds chia, especially in the content of calcium and potassium. However, chia has more phosphorus.

Finally, given the high content of fatty acids in *M. oleifera*, it has been compared with other products rich in these components that are consumed fresh (avocado) or dried (nuts) (Table 4). It should be noted that the dried leaves of *M. oleifera* far outweigh the avocado and walnut in terms of total omega-3 and the total content of polyunsaturated fatty acids (PUFA).

According to the WHO [60], total dietary fat should be between 15–30% of the total energy, of which less than 10% must be saturated fatty acids (SFA), between 6–10% must be polyunsaturated fatty acids (PUFA), and 15% must be monounsaturated fatty acids (MUFA). The ideal balance of SFA: MUFA: PUFA would be approximately 1:1.3:1 [61]. Within PUFAs, the most important are omega-6 and omega-3 fatty acids, with an omega-6 percentage of about 5–8% of the total dietary energy and between 1–2% of omega-3. Interest in omega-3 PUFAs has increased in recent years due to their various roles in promoting health and reducing the risk of cardiovascular disease and diabetes. These fatty acids are present in certain vegetables and soybeans and other plants have been genetically modified to contain higher levels of PUFA [62]. The most appropriate omega-6/omega-3 ratio is between 4/1 and 2/1, which favors a lower mortality rate in certain diseases [63]. If this ratio is exceeded, it can lead to health problems. Therefore, the challenge of today’s diet is to obtain, through consumption of food, lower ratios between these fatty acids [64]. However, it is not desirable to reduce the supply of omega-6 fatty acids because it would cause an imbalance in that proportion, but it is much more desirable to increase the supply of omega-3 fatty acids [63].

The dried leaf of *M. oleifera*, as shown in Table 4, has a high proportion of omega-3 fatty acids, which could be an alternative to enrich the food matrices in this component.

The composition of macronutrients and vitamins of groups B, A, C, and E in dried and fresh *M. oleifera* leaves is shown below in Table 5. As aforementioned, *M. oleifera* leaves contain nutritious compounds, highlighting the crude protein content of the dried leaf, which means that it is a good potential source of supplementary protein [59]. Calories, proteins, fats, carbohydrates, but especially vitamins and fiber, are greatly concentrated when drying *M. oleifera* leaves. Micronutrients are essential for human life. However, our body only requires minimal amounts of each to function properly. Contrary to what one might think, meeting each of the requirements of these micronutrients sometimes becomes complicated because of malnutrition problems, unbalanced diets, lack of education in food issues, and because they are in low doses in each of the foods [67].

The recommended daily amounts of vitamins are: 800 g for vitamin A, 12 mg for vitamin E, 80 mg for vitamin C, and 0.42 mg/1000 kcal for B vitamins [69]. In this regard, an intake of 11.43 g of fresh or 0.87–4.62 g of dried *M. oleifera* leaf would be sufficient to reach the recommended daily allowance of vitamin A since in the dried form, there is more fluctuation in values. To achieve the recommended daily allowance of vitamin E, an average of 2.68 g of fresh *M. oleifera* leaf and 11.11 g of dried *M. oleifera* leaf should be consumed. To reach the recommended daily allowance of vitamin C, between 36.36 and 154.74 g of fresh leaf and an average of 484.38 g of dried leaf should be consumed. Finally, for B vitamins, in a normal diet of 2000 kcal, 1400 g of fresh *M. oleifera* leaf should be consumed to reach the recommended daily allowance of vitamin B1, 1680 g for vitamin B2, and 105 g for vitamin B3. For the dried leaf of *M. oleifera*, 36.7 g for vitamin B1, 4.02 g for vitamin B2, and 10.65 g for vitamin B3 should be consumed.

Although fiber consumption is related to health benefits, its consumption in many countries is below the recommended amount (25 g/day in adults), since there is a greater supply and purchase of refined products. The food industry has a great opportunity to develop and to introduce in the market products with high content of fiber, which could also suppose a saving in the treatment of diseases [70]. However, the European Food Safety Authority (EFSA) recognized that higher than recommended amounts reduce the risk of coronary heart disease and type 2 diabetes and improve body weight maintenance [69]. In this case, 2777.78 g of fresh *M. oleifera* leaf or 207.47 g of dried *M. oleifera* leaf should be consumed to achieve the recommended daily allowance of fiber.

For all of the above, it is interesting to introduce *M. oleifera* in the food matrices in order to fortify their levels of some micronutrients to prevent deficiencies without modifying population eating habits [67].

*M. oleifera* has approximately 46 antioxidants, that is, it is one of the most powerful sources of natural antioxidants that help cells neutralize the effect of free radicals [71]. *M. oleifera* leaves are rich in flavonoids and also contain essential micronutrients with antioxidant activity or are directly related to this process, such as selenium or zinc [59].

The main present antioxidants are: quercetin, kaempferol, beta-sitosterol, caffeoylquinic acid, and zeatin [72]. It should be noted that the antioxidant power plays an important role in controlling the symptoms of the aging process and improving cardiovascular health. In addition, vitamin C and vitamin E present in *M. oleifera* also function as antioxidants [61]. In this sense, daily intake of *M. oleifera* will also be a contribution of antioxidants.

Scientists at the Asian Vegetable Research and Development Center (AVRDC) observed that boiled *M. oleifera* leaves or leaf powder provided at least three times more bioavailable iron than fresh *M. oleifera* leaves. Boiling also increased the antioxidant activity of the leaves. Furthermore, evidence of a higher nutrient content was recorded in mature leaves compared to young leaves although young shoots were preferred for consumption. Regarding vitamin A, it was higher during the warm and humid season. In contrast, iron and vitamin C were higher during the cold, dry season [32].

## 6. Medicinal Properties

Since time immemorial, humans have sought and known medicinal plants, since in addition to being used for the prevention of pathologies, they were easily accessible to the population, less aggressive for health, and with fewer side-effects. Therefore, they are considered an important tool for comprehensive health care.

*M. oleifera* is a plant with an important source of natural phytochemicals, which constitutes a basis for future research. Currently, there is a growing international interest in this tree, both in the field of food and in medicine and cosmetics [73].

All parts of *M. oleifera* (leaves and roots, seeds, bark, fruits, flowers, and immature pods) act as cardiac and circulatory stimulants. In addition, they have antitumor, antiulcer, antispasmodic, anti-inflammatory, and antidiabetic effects, helping to maintain normal blood sugar levels as they have a high content of ascorbic acid that aids in insulin secretion) [74]. Table 6 shows the main medicinal properties according to the organ of the plant consumed.

The aqueous extract of seeds and the fresh juice from *M. oleifera* leaf are effective against infectious skin bacteria such as *Staphylococcus aureus* and *Pseudomonas aeruginosa* [32]. The roots and seeds of *M. oleifera*, due to some active ingredients they contain, such as isothiocyanates and glucomoringin, are potent antibacterial and antifungal agents [78]. The *Moringin* alkaloid of the *M. oleifera* plant shows similarity to ephedrine in its activity [80]. Therefore, *M. oleifera* seeds have been shown to have a potential effect in the treatment of bronchial asthma [78]. Seed infusion has also been found to have antidiuretic properties [73].

Several bioactive compounds present in *M. oleifera* leaves such as nitrile, mustard oil glycosides, and thiocarbamate glycosides have a direct effect on blood pressure regulation. It is being used to treat different conditions in indigenous medicine, particularly in South Asia [26]. *M. oleifera* leaves contain sitosterol, a bioactive Phyto constitutive with a cholesterol-lowering effect [80]. The phenolic compounds present in the leaves give it a free radical scavenging property [80]. Leaf extracts have been shown to have antiproliferative effects, so they can inhibit the growth of cancer cells [22]. Moreover, these aqueous extracts regulate the hormone roid and can be used to treat hyperthyroidism and have an antioxidant effect [79]. Studies indicate that *M. oleifera* root has antiurolytic activity. One study also revealed an antilithic property of aqueous and alcoholic extracts from the root cortex. In fact, both extracts significantly reduced levels of urinary excretion and renal retention of oxalate, calcium, and phosphate [78].

## 7. Conclusions

To sum up, the cultivation of *M. oleifera* could be an alternative in places where climate change is preventing the development of traditional crops. Furthermore, as all parts of *M. oleifera* are edible (leaves, roots, flowers, pods, and seeds), they can be included in different food matrices, and therefore enrich their nutritional profile due to their high levels of proteins, fiber, vitamins, and antioxidants. It also has important nutritional properties such as anti-inflammatory, anti-asthmatic, cholesterol-lowering effect, antibacterial, and analgesic, among others. Its particular high value of omega 3 may help to formulate products with a recommended balance of omega-6/omega-3 ratio. *M. oleifera* also have many other possibilities to be used in the field of cosmetics, water purification, animal feed, fiber extraction for building material, and shell for biofuel.

Finally, as this plant is little known in developed countries, more information should be spread in order to enhance this crop and ensure different industrial sectors profit from all its parts, paving the way for a circular economy in a more sustainable way.

## Figures and Tables

**Table 1:** Nutritional value of fresh and dried *M. oleifera* leaves compared to other foods (Sources: [31,46]).

Content in (mg/100 g)	Fresh *M. oleifera* Leaves	Dry *M. oleifera* Leaves	Other Foods
Vitamin A	7	18.9	Carrot: 1.89
Vitamin C	220	17.3	Orange: 30
Calcium	440	2003	Cow’s milk: 120
Iron	085	28.2	Spinach: 1.14
Potassium	259	1324	Banana: 88
Protein	6700	27,100	Yogurt: 3100

**Table 2:** Amino acid content in dried *M. oleifera* leaf, soybeans, and beef (Sources: [48,57]).

Aminoacid Content (mg/100 g)	Dry *M. oleifera* Leaf	Soy	Beef
Arginine	1325	380	1118
Histidine *^1^	613	221	603
Lysine *	1325	233	1573
Tryptophan *	425	103	---------
Phenylalanine *	1388	708	778
Methionine *	350	296	478
Threonine *	1188	328	812
Leucine *	1950	1764	1435
Isoleucine *	825	803	852
Valine *	1063	728	886

* Essential amino acids/*^1^ essential amino acid only during childhood.

**Table 3:** Composition of minerals in dried and fresh *M. oleifera* leaf, spinach, and chia (Sources: [48,58,59].

Minerals (mg/100 g)	Fresh *M. oleifera* Leaf	Spinach	Dry *M. oleifera* Leaf	Chia
Calcium	440	117	2003	631
Iron	0.85	2.7	28.2	0.01
Copper	0.07	-----	0.57	-----
Magnesium	42	-----	368	335
Phosphorus	70	46	204	860
Potassium	259	554	1324	407
Zinc	0.16	-----	3.29	-----

**Table 4:** Composition of fatty acids in the dried leaf of *M. oleifera* (Sources: [59,65,66]).

Fatty Acids (%)	Dried *M. oleifera* Leaves	Avocado	Walnut
Caprylic	0.07	-----	2.28
Lauric	0.58	-----	-----
Myristic	3.66	0.12–0.13	-----
Palmitic	11.79	19.7	11.56
Palmitoleic	0.17	13.49	-----
Margaric	3.19	-----	-----
Stearic	2.13	1.51–1.52	1.14
Oleic	3.96	49.55	11.37
Linoleic	7.44	14.01	18.91
Linolenic	44.57	1.26	3.41
Total de Omega-6	7.64	14.01	18.91
Total de Omega-3	44.57	1.26	3.41
MUFA (total monounsaturated fatty acids)	4.48	63.53	14.2
PUFA (total polyunsaturated fatty acids)	52.21	15.27	23.2
Omega-6/Omega-3 Ratio	0.17	11.12	5.54
MUFA:PUFA Relationship	0.09	4.16	0.61

**Table 5:** Composition of macronutrients and vitamins of group B, A, C, and E in dried and fresh leaves of *M. oleifera* (Sources: [31,48,67,68]).

Nutritional Analysis	Fresh *M. oleifera* Leaves (per 100 g)	Dry *M. oleifera* Leaves (per 100 g)
Humidity (%)	75–79.7	4.8–7.5
Calories (Kcal)	49.5–92	205–329
Proteins (g)	5.5–9.4	27.1–33.5
Fats (g)	1.4–2.1	2.3–9.75
Carbohydrates (g)	8.633–13.4	38.2–41.2
Fiber (g)	0.9	7.48–30.97
Vitamin B1 (mg)	0.06	2.02–2.64
Vitamin B2 (mg)	0.05	20.5–21.3
Vitamin B3 (mg)	0.8	7.6–8.2
Vitamin E (mg)	448	108–113
Vitamin A (mg)	7	17.3–91.8
Vitamin C (mg)	51.7–220	15.8–17.3

**Table 6:** Main medicinal properties according to the part of *M. oleifera* consumed.

Part of the Plant	Medicinal Use	References
Roots	Analgesic, anti-inflammatory, antitumor, antidiabetic, snake bite, antiulcer, antispasmodic, cholesterol-lowering effect, antibacterial, antiurolytic, antifungal, antidiuretic and antihypertensive.	[22,74,75,76,77,78]
Leaves	Anticatarrhal, antidiabetic, antiscurb, antihypertensive, antiproliferative, antioxidant, anxiolytic, diuretic, pharyngitis, cholesterol-lowering effect, hemorrhoids, glandular swellings, anti-inflammatory and anti-hyperthyroidism.	[22,74,78,79]
Flowers	Anti-inflammatory, antipsychotic and anti-tumor.	[22,74,78]
Seeds	Antidiuretic, antitumor, genitourinary, antituberculous, anti-asmatic, antibacterial and hepatoprotective.	[22,32,74,75,76]
